# Opinions of the Profession Concerning the “Reform School”

**Published:** 1859-09

**Authors:** 


### EDITORIAL.

### OPINIONS OF THE PROFESSION CONCERNING THE "REFORM SCHOOL."

We have taken pains to mark every notice of the medical department of the Lind University which has fallen under our observation since the issue of the circular of the proposed school. Thus far its reception has not been flattering. The little axe carried down to Louisville to be ground was rejected; the prospect at Decatur was so poor that no attempt was made to get an endorsement directly, and an indirect one to get a member of the faculty recommended as a candidate for President of the American Medical Association next year signally failed, the society thinking, apparently, that there might be a man in all New England capable of filling such an office.

Among the medical journals few seem to have regarded it as deserving of comment. The exceptions are the New York Monthly *Review*, the New Orleans *Medical News and Hospital Gazette*, the Cleveland *Medical Gazette*, and the Nashville Monthly *Record*.

The first of these, edited by Austin Flint, jr., who, to alter a proverbial phrase a little, may be said to be "a spark from the old stove," has the following paragraph respecting it:

"Prof. N. S. Davis, formerly the editor of the Chicago *Medical Journal*, who was connected also with the Rush Medical College, withdrew, with several of the faculty, and established the Lind University upon the principle which Prof. Davis has so long advocated. Whether it will be a success remains to be seen; we regard as extremely doubtful both its success and its benefit to the cause of medical education."

The New Orleans journal, edited by Drs. Brickell and Fenner, intimates its doubts gently, as follows:

"We notice that there are eleven professorships, and there are junior and senior departments, in the first of which the students attend the lectures on five branches, besides dissecting; and the second they attend lectures on six branches, besides clinical lectures and dissecting. All our readers know we are the strong and practical advocates for improvement in medical teaching, and we hope this scheme may succeed, but we can't exactly see through it. We fear the matter is a little too complicated."

Prof. Weber, of the Cleveland *Medical Gazette*, which we have already announced, makes the following remarks, full of force, concerning it, after giving the plan of instruction:

"Now it requires a good deal of imagination to be able to compare this new mode of teaching in the Lind University with the systematic training of the medical student in the old schools of Europe, where eight terms, and sometimes ten, are required, and the student hears lectures on one subject not only *once*, but two or three times over again.

"If the medical student of our country is to learn his profession by listening to lectures; *one course* on one particular subject, ever so ably and minutely delivered, and regularly attended, will not qualify him to understand the subject completely. It requires *time* and *study* to comprehend *any* of the branches of the medical sciences thoroughly. Ever since we have been connected with medical teaching we have felt the deficiency of our present system, and we will hail the time when all the institutions of our land shall raise the banner of TRUE IMPROVEMENT in the great cause of medical education. But we must have an entire revolution of the present condition of things; a little stirring, a little change will not benefit the student nor the profession, nor those who brought this change about—*experientia docebit.*"

Prof. Wright, of the Nashville Monthly *Record* for July, goes more into details. His objections may be enumerated as follows:

1. Of the list of Professors' names paraded, one is an Emeritus, and two others are given twice, so that this pretended increase is a deception.

2. General pathology and physiology should be taught by the same chair. It is a mistake to separate them. Prof. Wright may justly think the chair of public hygiene superfluous.

3. Assigning medical jurisprudence to an aged gentleman, instead of a physician, renders that chair practically of little value, as the principal facts of the science are found in the fields of chemistry, anatomy, physiology, obstetrics, etc.

4. The division of surgery into two parts is objected to.

Prof. Wright also makes an objection to the fees, which we deem less feasible than each of the former, which are all well founded.

These are all the notices of the "reform school" presented in our exchanges. Diminishing the number of lectures and the number of terms required is evidently not regarded as "reform."

